# Patient-Related Factors Associated with Adverse Outcomes Following Weaning from Veno-Arterial Extracorporeal Membrane Oxygenation

**DOI:** 10.3390/jcm12237406

**Published:** 2023-11-29

**Authors:** Marius Keller, Henning Gloeckner, Sibel Sari-Yavuz, Helene A. Haeberle, Christian Schlensak, Peter Rosenberger, Harry Magunia, Michael Koeppen

**Affiliations:** 1 Department of Anesthesiology and Intensive Care Medicine, Eberhard Karls University, Hoppe-Seyler-Str. 3, 72076 Tuebingen, Germany; henning.gloeckner@icloud.com (H.G.); sibel.sari@med.uni-tuebingen.de (S.S.-Y.); helene.haeberle@med.uni-tuebingen.de (H.A.H.); peter.rosenberger@uni-tuebingen.de (P.R.); harry.magunia@med.uni-tuebingen.de (H.M.); michael.koeppen@med.uni-tuebingen.de (M.K.); 2 Department of Thoracic and Cardiovascular Surgery, Eberhard Karls University, Hoppe-Seyler-Str. 3, 72076 Tuebingen, Germany; christian.schlensak@med.uni-tuebingen.de

**Keywords:** extracorporeal membrane oxygenation, veno-arterial, extracorporeal life support, mechanical circulatory support, weaning, weaning fail

## Abstract

Background: Veno-arterial extracorporeal membrane oxygenation (vaECMO) removal reflects a critical moment and factors of adverse outcomes are incompletely understood. Thus, we studied various patient-related factors during vaECMO removal to determine their association with outcomes. Methods: A total of 58 patients from a university hospital were included retrospectively. Demographic, clinical, and echocardiographic parameters were recorded while under vaECMO support, as well as the need for inotropic and vasoactive-inotropic scores (VIS). Successful weaning was defined as 28-day survival without reinitiation of vaECMO. Results: Patient age differed significantly between patients with a successful and a failed vaECMO weaning (54 ± 14 vs. 62 ± 12 years, *p* = 0.029). In univariable logistic regression, age (OR 0.952 (0.909–0.997), *p* = 0.038), the necessities for inotropic agents at the time of echocardiography (OR 0.333 (0.113–0.981), *p* = 0.046), and vaECMO removal (OR 0.266 (0.081–0.877), *p* = 0.030) as well as the dobutamine dose during removal (OR 0.649 (0.473–0.890), *p* = 0.007), were significantly associated with a successful weaning from vaECMO. Age (HR 1.048 (1.006–1.091), *p* = 0.024) and the VIS (HR 1.030 (1.004–1.056), *p* = 0.025) at the time of vaECMO removal were independently associated with survival in bivariable Cox regression. In Kaplan–Meier analysis, a VIS of >5.1 at vaECMO removal was associated with impaired survival (log-rank *p* = 0.025). Conclusions: In this cohort, age and the extent of vasoactive-inotropic agents were associated with adverse outcomes following vaECMO, whereas echocardiographic biventricular function during vaECMO support was not.

## 1. Introduction

In the case of refractory circulatory failure, the initiation of veno-arterial extracorporeal membrane oxygenation (vaECMO, also known as extracorporeal life support) is utilized as a last resort to establish sufficient organ perfusion [1,2]. Weaning from vaECMO becomes a priority once the detrimental effects of shock have been overcome and cardiac function has been restored. After achieving hemodynamic stabilization, the ideal moment of the discontinuation and removal of vaECMO—if feasible—frequently proves challenging in clinical practice [3]. Subsequently, obtaining real-world data is crucial in revealing the patients’ risk factors and hemodynamic characteristics that may lead to unfavorable outcomes post-vaECMO therapy discontinuation [4,5]. Possible candidate indices include echocardiographic functional parameters, the specific need for vasoactive and inotropic agents to maintain adequate hemodynamics, comorbidities—such as concomitant renal failure—and the presence of vaECMO-related complications. Despite standardized weaning trials which aim for the evaluation of the patients’ capacities to maintain adequate hemodynamics after the termination of circulatory support [6], the short-term prognosis may be influenced by variables that go beyond those measured in weaning trials [7,8]. Therefore, this study examined various clinically available patient-related parameters and their associations with unfavorable outcomes post-vaECMO removal in a retrospective patient cohort from a university hospital.

## 2. Materials and Methods

### 2.1. Study Design and Ethical Approval

The study was conducted as a monocentric retrospective observational cohort study among patients who underwent vaECMO therapy at the intensive care unit (ICU) operated by the Department of Anesthesiology & Critical Care Medicine of Eberhard Karls University, Tuebingen, Germany. The ICU has a maximum capacity of 40 patients and treats all surgical intensive care cases of the University Hospital Tuebingen. The Department of Thoracic and Cardiovascular Surgery is available in-house 24/7. Implantation of vaECMO is usually performed by the institutional cardiac surgery team and supported by the ICU team, e.g., for respiratory support and transesophageal echocardiographic guidance during cannulation. The local ethics committee approved the study on 23 August 2019 (IRB #588/2019BO2). Informed consent was waived by the board due to the pseudonymized handling of retrospective data in accordance with German privacy regulations. A two-year period was selected to achieve homogenous data. During this study period, no changes in treatment protocols were performed.

### 2.2. Patient Selection

Retrospective patient screening between January 2017 and December 2018 was performed using the ICU’s institutional electronic database. Patients were eligible for inclusion if they received vaECMO therapy, were >18 years old, and underwent echocardiography within 48 h prior to the removal of vaECMO. Only patients with complete clinical datasets and sufficient image quality to perform echocardiographic measurements were included. If vaECMO support was discontinued within palliative care, the patient was excluded from the final analysis.

### 2.3. Clinical and Hemodynamic Parameters

Demographic and clinical characteristics were extracted from the digital patient records. Arterial hypertension and chronic lung disease were regarded as present if appropriate drugs were taken regularly prior to hospitalization. The Simplified Acute Physiology Score (SAPS) II at ICU admission was calculated according to the published formula [9]. Chronic kidney disease was defined if the pre-admission-estimated glomerular filtration rate was below 60 mL/min. The primary indications for vaECMO initiation were extracted from digitally stored reports.

The necessity for inotropic agents was defined as the administration of an inotropic drug equal to or higher than the minimum maintenance dose according to the official prescribing information (epinephrine: 0.05 µg/kg/min; dobutamine: 2 µg/kg/min; milrinone: 0.375 µg/kg/min; levosimendan: 0.05 µg/kg/min). In case of altered vascular reactivity, the stepwise institutional vasopressor regimen includes norepinephrine up to a dose of 0.3 µg/kg/min, followed by additional vasopressin administration. The vasoactive-inotropic score was calculated according to the published formula [10]: VIS = dobutamine dose [µg/kg/min] + 100 × epinephrine dose [µg/kg/min] + 100 × norepinephrine dose [µg/kg/min] + 10 × milrinone dose [µg/kg/min] + 10,000 × vasopressin dose [U/kg/min].

### 2.4. Echocardiography

Patients’ echocardiograms were stored in the institutional digital database and accessed with a specialized software interface (IntelliSpace Cardiovascular, Release 6.1, Philips Healthcare, Inc., Andover, MA, USA). Commercially available ultrasound systems including high-resolution two-dimensional probes (Philips Healthcare, Inc., Andover, MA, USA) were used for transthoracic and transesophageal echocardiography. Echocardiography studies were included only if they were conducted within 48 h before the removal of vaECMO and contained baseline imaging. The baseline echocardiographic function was quantified in recordings at the currently established vaECMO flow rates without experimental flow reductions. Hence, baseline echocardiographic parameters quantify cardiac function under “full” pre- and afterload alterations associated with vaECMO. The acoustic window (transesophageal/transthoracic) and the current vaECMO flow rates during echocardiographic evaluation were extracted for each patient. Left ventricular (LV) and right ventricular (RV) dimensions, LV volumes/ejection fraction (EF, Simpson method), and RV fractional area change (FAC) were quantified offline within the IntelliSpace Cardiovascular software package. LV and RV global longitudinal strain (GLS) were measured offline using dedicated software (2D Cardiac Performance Analysis, Version 1.2.0.24, Tomtec, Unterschleissheim, Germany). All echocardiographic measurements were performed by experienced investigators according to guideline recommendations [11].

### 2.5. Endpoint Definition

Survival times were extracted from electronic patient records using the institutional database. Weaning from vaECMO was defined as successful if patients survived 28 days post-vaECMO removal and did not require reinitiation of extracorporeal circulatory support. Otherwise, vaECMO weaning was regarded as failed. Mid- and long-term survival data were extracted if patients presented to follow-up appointments at any institution of University Hospital Tuebingen.

### 2.6. Statistical Analysis

Samples were tested for normal distribution using the D’Agostino–Pearson test. Normally distributed variables are expressed as the mean ± standard deviation and as the median (interquartile range) in the case of a non-normal distribution. Normally distributed variables were compared using unpaired Student’s *t*-test, whereas non-normally distributed variables were compared with the Mann–Whitney U test. Differences in proportions between the two groups were tested for significance using the Chi-squared test. Univariable logistic regression was performed for a binomial endpoint and the variables’ results are expressed as odds ratio (OR), their 95% confidence interval (CI), and *p*-value. Univariable and multivariable Cox regression was used to analyze the variables’ associations with survival, and the results are expressed as hazard ratio (HR), 95% CI, and *p*-value. Receiver operating characteristics analysis was performed to determine the area under the curve and the ideal cutoff value of variables following regression analysis. Kaplan–Meier analysis and the log-rank test were used to compare survival between the two groups. Prism 9 (GraphPad Software, San Diego, CA, USA) and MedCalc (MedCalc Software Ltd., Version 13.0.4.0, Ostend, Belgium) were used for statistical analyses. *p*-values <  0.05 are considered statistically significant.

## 3. Results

### 3.1. Baseline Patient Demographics and Clinical Characteristics

After retrospective patient screening for eligibility, 58 patients with complete datasets could be included in the final analysis. The baseline demographic and clinical characteristics of the study cohort are listed in Table 1. Patients successfully weaned from vaECMO (*n* = 33; 58%) were significantly younger than patients with failed weaning. However, the two outcome groups did not differ significantly in disease severity upon admission or comorbidities. As 28-day mortality was part of the composite endpoint of successful weaning from vaECMO, these patients unsurprisingly showed reduced mortalities compared to patients of the unfavorable outcome group. Early deaths explain the significantly shorter ICU stays of patients in whom weaning was unsuccessful. Regarding vaECMO treatment, the two outcome groups did not present with significant differences in the indication for circulatory support, durations of mechanical ventilation prior to vaECMO implantation, the need for renal replacement therapy or vaECMO-associated complications (Table 2). In trend, however, fewer patients with successful vaECMO weaning suffered from bleeding complications (24% vs. 52%, *p* = 0.055).

### 3.2. Vasoactive-Inotropic Agents and Echocardiography

**Figure 1:**
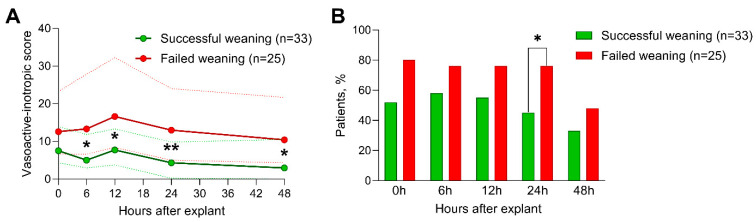
Necessity for inotropic agents and vasoactive-inotropic scores following vaECMO weaning. Time courses of the necessity for inotropic agents (**A**) and the vasoactive inotropic scores (**B**) after vaECMO removal of patients with successful weaning (green) versus patients who failed weaning (red). (**A**) shows the medians (bold lines) and interquartile ranges (fine dotted lines) and (**B**) shows the percentages (bars). *, *p* < 0.05; **, *p* < 0.01.

Table 3 lists the echocardiographic parameters under full flow rates before weaning from vaECMO. Overall, most patients underwent transesophageal echocardiography, whereas the remaining patients were evaluated with transthoracic echocardiography. The vaECMO flow rates at the time of the baseline echocardiograms were similar between the outcome groups. Strikingly, none of the investigated echocardiographic measures under vaECMO support were significantly different between patients with a successful and failed weaning from vaECMO, including left and right ventricular volumes, LV EF, LV GLS, and RV GLS. The vasoactive and inotropic agents administered are reported in Table 4. Similarly, at the time of the echocardiographic evaluation and vaECMO removal, the necessity of inotropics as well as the extent of vasoactive–inotropic agents (reflected by VIS values) were comparable in both outcome groups. However, dobutamine doses during vaECMO removal were significantly lower in patients with a favorable outcome. During the following 48 h, however, patients whose weaning was declared successful required significantly fewer vasoactive-inotropic agents to maintain adequate hemodynamics 6 h, 12 h, 24 h, and 48 h post-removal (Figure 1A). Furthermore, a significantly higher percentage of patients in the unfavorable outcome group required inotropics 24 h after vaECMO weaning (45% vs. 76%, *p* = 0.036, Figure 1B).

### 3.3. Association with Outcome

**Figure 2:**
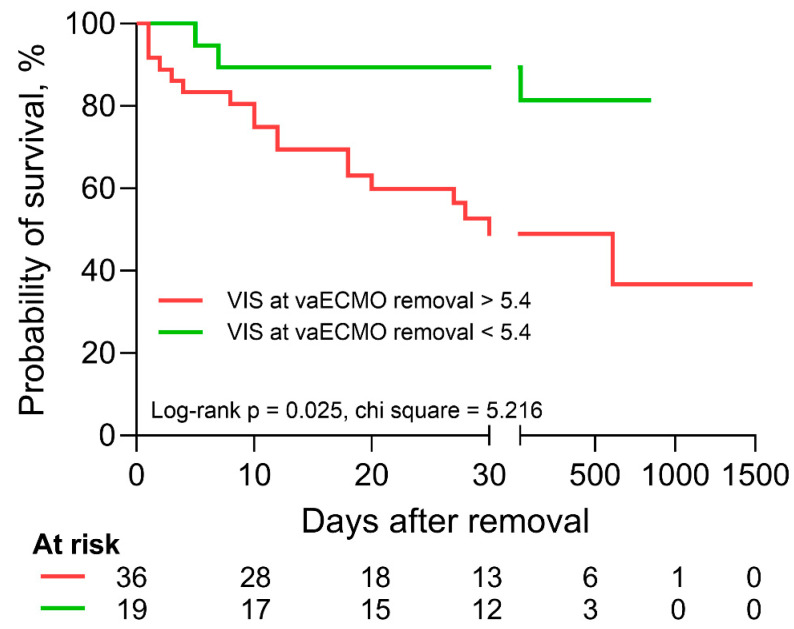
Kaplan–Meier analysis and log-rank test with regard to vasoactive-inotropic score (VIS) at the time of vaECMO removal. Patients with a VIS of greater than 5.4 (red) at the time of vaECMO removal experienced significantly reduced survival according to Kaplan–Meier analysis and log-rank test.

In univariable logistic regression, age (OR 0.952 (0.909–0.997), *p* = 0.038), the necessities for inotropic agents at the time of the echocardiographic evaluation (OR 0.333 (0.113–0.981), *p* = 0.046) and vaECMO removal (OR 0.266 (0.081–0.877), *p* = 0.030), as well as the dobutamine dose during removal (OR 0.649 (0.473–0.890), *p* = 0.007), were significantly associated with successful weaning from vaECMO (Table 5). In univariable Cox regression analysis, age (HR 1.045 (1.006–1.086), *p* = 0.025), the VIS (HR 1.029 (1.003–1.055), *p* = 0.027) and the dobutamine dose (HR 1.232 (1.036–1.466), *p* = 0.029) at time of vaECMO removal were significantly associated with survival following the removal of vaECMO (Table 6). In a bivariable Cox regression model, age and the VIS at the time of vaECMO removal were shown to be independently associated with survival (age: HR 1.048 (1.006–1.091), *p* = 0.024; VIS at the time of vaECMO removal: HR 1.030 (1.004–1.056), *p* = 0.025). In receiver operating characteristics analysis, the VIS at the time of vaECMO removal had an area under the curve of 0.681 (0.546–0.798, *p* = 0.010). A cutoff value of 5.4 was defined for ideal discrimination and the two patient groups showed significantly altered survival in Kaplan–Meier analysis (Figure 2). A follow-up exceeding 28 days after vaECMO removal was available in *n* = 26 patients.

## 4. Discussion

The aim of the present study was to identify patient-related factors linked with unfavorable outcomes after the discontinuation and removal of vaECMO support. We performed a retrospective analysis of patients undergoing vaECMO at our intensive care unit and included various demographic, clinical, and echocardiographic parameters. A 28-day vaECMO-free composite endpoint was utilized to delineate two outcome groups, potentially overcoming the limitations associated with the discrepancies of using weaning success and survival as single endpoints [3]. Our findings indicate that older patients are at a higher risk of unsuccessful vaECMO weaning and comprised survival. To our surprise, we found no specific echocardiographic parameters quantified under full vaECMO support within 48 h prior to vaECMO removal which were associated with the endpoint. Nevertheless, the use of inotropic agents and the extent of vasoactive-inotropic support were identified to be associated with successful weaning from vaECMO and survival.

Although the use of vaECMO has risen as part of a multimodal approach to treat cardiocirculatory collapse [12], several factors remain unresolved. Although the impact of vaECMO treatment on survival appears to depend on the underlying indication [13,14], the optimal timing for weaning from vaECMO remains unclear. Our findings and previous trials provide evidence that several patient-related factors are indicative of adverse outcomes following the discontinuation and removal of vaECMO.

Patient age at the time of weaning is a well-known contributor to outcome [15,16]. Our results confirm previous findings and support the body of evidence. Although the primary reason for the initiation of vaECMO support was previously shown to influence prognosis [17], the indications for vaECMO did not differ between patients with good and poor outcomes in the present study. This is particularly of interest as patients after extracorporeal cardiopulmonary resuscitation were equally distributed in both outcome groups, whereas this group of patients is known to be at a high risk of in-hospital death [18]. Regarding pre-existing comorbidities, a history of ischemic or coronary heart disease was associated with poor outcomes in a recent study by Cusanno et al. [19] but did not impact vaECMO weaning success in our patients.

Among the most significant factors associated with unsuccessful discontinuation from the extracorporeal circuit were the administration of positive inotropic drugs and vasopressors. As there were no differences in the underlying cause for vaECMO initiation, it appears unlikely that this variation was due to differences in pathology. Thus, the need for inotropes and vasopressors may more likely indicate persistent myocardial impairment and altered vascular reactivity. Our findings are in line with other studies and support the hypothesis that the necessity for vasoactive and/or inotropic agents can serve as a predictor of successful/unsuccessful weaning from vaECMO [20,21]. A recent systematic review and meta-analysis found that successful weaning is associated with specific hemodynamic, laboratory, and echocardiographic parameters, such as higher pulse and mean blood pressures, lower creatinine and lactate levels, and higher LV EF [7].

Early on, Aissaoui et al. identified multiple echocardiographic parameters that could guide clinical decision-making regarding the weaning of vaECMO [22]. They found that the velocity–time integral in the left ventricular outflow tract as well as the tissue Doppler-derived lateral mitral annulus peak systolic velocity predict a positive outcome when used under standardized weaning trial conditions. Although the current body of literature suggests that echocardiographic parameters during weaning trials are predictive of adverse outcomes following vaECMO removal [23,24,25,26,27], the significance of echocardiographic evaluations under full vaECMO support requires further investigation [28]. Despite being limited to a retrospective dataset, we assessed conventional and innovative echocardiographic measurements under the currently established vaECMO flow rates and within 48 h prior to vaECMO removal to determine their utility for this purpose. Strikingly, we could not identify any echocardiographic markers under baseline conditions—including LV and RV GLS—to be associated with vaECMO weaning success in our cohort. This potentially translates into substantial clinical implications: biventricular echocardiographic functional parameters measured under hemodynamic support of vaECMO may have low predictive value, highlighting the need for standardized weaning trials including echocardiographic measurements under flow rate reductions. Unfortunately, systematic echocardiographic weaning trials were not part of the clinical management strategy at our institution during the study period.

Furthermore, sequential organ failure assessment scores at the time of weaning also serve as a predictor for successful weaning from vaECMO since this score summarizes dysfunction across multiple organ systems [20]. We found no differences between the two groups using SAPS II in our study, suggesting that even when other organ systems may be functioning adequately, impairment of cardiac function still predicts outcomes. Other authors have suggested that patients undergoing vaECMO often receive invasive simultaneous mechanical ventilation, thus impairing lung function due to mucociliary dysfunction [29]. Advocates call for a systemic evaluation of the bronchial system by bronchoscopy, since cardiorespiratory conditions at the time of weaning were suboptimal in patients who did not wean successfully [30]. In our study, we did not assess pulmonary function at the time of vaECMO weaning; thus, we cannot confirm whether this was a contributing factor in our cohort.

### Limitations

The retrospective and monocentric nature of this study, as well as the small sample size, limits the objectivity of the results. Hence, the generalizability of our findings remains unclear. A major limitation of our report is the lack of standardized weaning protocols during the study period, which may have led to the inappropriate weaning of patients with inadequate biventricular function. Many key parameters were not routinely quantified in most patients at the desired time-points and could therefore not be analyzed, such as biomarkers or echocardiographic indices of diastolic function and cardiac output. Invasive hemodynamic parameters were not systematically available in this cohort, thus hindering the investigation of their impact on patient outcomes. Additionally, the low proportion of patients included in the final analysis suggests the potential for significant inclusion bias. The high number of patients excluded due to palliative vaECMO removal might be caused by patients primarily implanted during earlier stages of extracorporeal circulatory support strategies, in whom vaECMO implantation would be avoided according to current standards (e.g., due to old age, irreversible brain damage, no goal of care).

## 5. Conclusions

Collectively, our findings indicate that patients who are older and require high doses of inotropics and/or vasopressors to maintain hemodynamic stability face increased risks of adverse outcomes following the termination of vaECMO therapy. Furthermore, echocardiographic functional parameters measured before weaning under full vaECMO support carry no incremental information with regard to weaning success.

## Figures and Tables

**Table 1:** Patient characteristics of the study cohort.

	All Patients (*n* = 58)	Successful Weaning (*n* = 33)	Failed Weaning (*n* = 25)	*p*-Value
Demographics				
**Percentage of total, %**	100	57	43	-
**Age, years**	57 ± 14	54 ± 14	62 ± 12	**0.029**
**Female sex, *n***	17 (29%)	9 (27%)	8 (32%)	0.902
**Body mass index (kg/m^2^)**	27.2 (23.9–29.4)	25.8 (23.8–28.8)	27.8 (24.8–30.3)	0.120
Disease severity and comorbidities at ICU admission				
**SAPS II**	47 ± 12	49 ± 12	45 ± 12	0.242
**Chronic lung disease, *n***	3 (5%)	2 (6%)	1 (4%)	0.796
**Coronary artery disease, *n***	19 (33%)	9 (27%)	10 (40%)	0.445
**Arterial hypertension, *n***	30 (52%)	17 (52%)	13 (52%)	0.791
**Chronic kidney disease, *n***	5 (9%)	5 (15%)	0 (0%)	0.121
**History of stroke, *n***	4 (7%)	1 (3%)	3 (12%)	0.414
Outcome				
**ICU length of stay, days**	27 ± 16	30 ± 19	24 ± 11	0.151
**ICU mortality, *n***	22 (38%)	2 (6%)	20 (80%)	**<0.001**
**In-hospital mortality, *n***	23 (40%)	2 (6%)	21 (84%)	**<0.001**
**14-day mortality, *n***	15 (26%)	0 (0%)	15 (60%)	**<0.001**
**28-day mortality, *n***	22 (38%)	0 (0%)	22 (88%)	**<0.001**
**Survival post-removal, days**	27 (10–262)	175 (27–558)	10 (2–19)	**<0.001**

Values are means ± standard deviations, medians (interquartile ranges), or *n* (%). Significant *p*-values are highlighted in bold. ICU, intensive care unit; SAPS, Simplified Acute Physiology Score.

**Table 2:** Information on vaECMO treatment.

	All Patients (*n* = 58)	Successful Weaning (*n* = 33)	Failed Weaning (*n* = 25)	*p*-Value
Indications for vaECMO support				
**Acute ischemic or valvular cardiogenic shock, *n***	29 (50%)	17 (52%)	12 (48%)	0.875
**Decompensated chronic heart failure, *n***	1 (2%)	1 (3%)	0 (0%)	0.207
**Severe pulmonary artery embolism, *n***	1 (2%)	1 (3%)	0 (0%)	0.207
**eCPR, *n***	27 (46%)	14 (42%)	13 (52%)	0.624
Treatment during vaECMO support				
**Mechanical ventilation prior to vaECMO implantation, *n***	32 (55%)	16 (49%)	16 (64%)	0.384
**Mechanical ventilation at the time of vaECMO removal, *n***	43 (74%)	23 (70%)	20 (80%)	0.576
**Total vaECMO run time, days**	9 (5–13)	7 (5–11)	10 (8–14)	0.093
**Renal replacement therapy total, *n***	26 (45%)	13 (39%)	13 (52%)	0.472
**Renal replacement therapy at the time of vaECMO removal, *n***	17 (29%)	9 (27%)	8 (32%)	0.0902
Complications of vaECMO support				
**Hemorrhagic complications, *n***	21 (36%)	8 (24%)	13 (52%)	0.055
**Received blood transfusions, *n***	55 (95%)	30 (91%)	25 (100%)	0.348
**Limb ischemia, *n***	1 (2%)	1 (3%)	0 (0%)	0.881

Values are medians (interquartile ranges) or *n* (%). eCPR, extracorporeal cardiopulmonary resuscitation; vaECMO, veno-arterial extracorporeal membrane oxygenation.

**Table 3:** Echocardiographic assessment within 48 h prior to vaECMO removal.

	All Patients (*n* = 58)	Successful Weaning (*n* = 33)	Failed Weaning (*n* = 25)	*p*-Value
**Transesophageal echocardiography, *n***	36 (62%)	17 (52%)	19 (76%)	0.111
**vaECMO flow at time of echocardiography, L/min**	3.1 ± 1.0	3.0 ± 1.0	3.2 ± 1.0	0.357
**LV IDd, mm**	49 ± 9	50 ± 11	48 ± 8	0.555
**LV EDV, mL**	139 ± 61	135 ± 55	145 ± 70	0.552
**LV EDVi, mL/m^2^**	70 ± 31	70 ± 29	70 ± 35	0.988
**LV ESV, mL**	92 ± 46	89 ± 42	97 ± 52	0.543
**LV ESVi, mL/m^2^**	47 ± 24	46 ± 22	47 ± 26	0.933
**LV GLS, %**	−8.8 ± 5.2	−8.9 ± 5.5	−8.8 ± 4.9	0.922
**LV EF, %**	35 ± 12	35 ± 12	34 ± 13	0.773
**RV IDd basal, mm**	35 ± 8	34 ± 9	37 ± 8	0.169
**RV IDd, mid, mm**	27 ± 7	26 ± 7	28 ± 7	0.208
**RV length, mm**	70 ± 15	70 ± 16	70 ± 13	0.895
**RV FAC, %**	33 ± 10	32 ± 9	35 ± 10	0.189
**RV GLS, %**	−12.1 ± 6.0	−11.5 ± 5.6	−12.9 ± 6.6	0.382

Values are means ± standard deviations or *n* (%). EDV(i), end-diastolic volume (index); EF, ejection fraction; ESV(i), end-systolic volume (index); FAC, fractional area change; GLS, global longitudinal strain; LV, left ventricular; IDd, internal dimension end-diastolic; RV, right ventricular; vaECMO, veno-arterial extracorporeal membrane oxygenation.

**Table 4:** Hemodynamic agents.

	All Patients (*n* = 58)	Successful Weaning (*n* = 33)	Failed Weaning (*n* = 25)	*p*-Value
**VIS** **at time of echocardiography**	5.0 (1.7–12.7)	5.0 (0–13.0)	6.8 (4.0–14.1)	0.201
**Necessity of inotropic agents at time of echocardiography, *n***	26 (45%)	11 (33%)	15 (60%)	0.075
**VIS** **at time of vaECMO removal**	11.9 (5.0–18.3)	7.5 (3.1–17.1)	12.6 (6.7–21.8)	0.107
**Necessity of inotropic agents at time of vaECMO removal, *n***	37 (64%)	17 (52%)	20 (80%)	0.054
**Norepinephrine dose at the time of vaECMO removal, µg/kg/min**	0.07 (0–0.14)	0.03 (0–0.13)	0.08 (0.02–0.14)	0.254
**Dobutamine dose at the time of vaECMO removal, µg/kg/min**	0 (0–2.5)	0 (0–0)	0 (0–5.0)	**0.008**
**Milrinone dose at the time of vaECMO removal, µg/kg/min**	0.26 (0–0.50)	0.25 (0–0.47)	0.26 (0–0.50)	0.811
**VIS** **6 h post-removal**	9.4 (3.1–20.0)	5.0 (2.3–14.1)	13.3 (6.9–26.7)	**0.016**
**Necessity of inotropic agents 6 h post-removal, *n***	38 (66%)	19 (58%)	19 (76%)	0.250
**VIS** **12 h post-removal**	10.4 (3.8–23.6)	7.1 (3.1–16.6)	16.6 (8.5–31.8)	**0.015**
**Necessity of inotropic agents 12 h post-removal, *n***	37 (64%)	18 (55%)	19 (76%)	0.169
**VIS** **24 h post-removal**	8.0 (1.8–18.0)	4.3 (0–11.6)	13.0 (5.0–23.9)	**0.009**
**Necessity of inotropic agents 24 h post-removal, *n***	34 (59%)	15 (45%)	19 (76%)	**0.036**
**VIS** **48 h post-removal**	5.9 (0–14.5)	3.0 (0–11.7)	10.4 (4.5–20.3)	**0.021**
**Necessity of inotropic agents 48 h post-removal, *n***	23 (40%)	11 (33%)	12 (48%)	0.376

Values are medians (interquartile ranges) or *n* (%). Significant *p*-values are highlighted in bold. vaECMO, veno-arterial extracorporeal membrane oxygenation; VIS, vasoactive-inotropic score.

**Table 5:** Univariable logistic regression for successful weaning from vaECMO.

	OR (95% CI)	*p*-Value
**Age, years**	0.952 (0.909–0.997)	**0.038**
**SAPS II at admission**	1.027 (0.982–1.074)	0.241
**VIS** **at time of echocardiography**	0.974 (0.920–1.031)	0.358
**Inotropic agents at time of echocardiography**	0.333 (0.113–0.981)	**0.046**
**VIS** **at time of vaECMO removal**	0.975 (0.938–1.013)	0.193
**Inotropic agents at time of vaECMO removal**	0.266 (0.081–0.877)	**0.030**
**Dobutamine dose at the time of vaECMO removal**	0.649 (0.473–0.890)	**0.007**

Significant *p*-values are highlighted in bold. CI, confidence interval; OR, odds ratio; SAPS, Simplified Acute Physiology Score, vaECMO, veno-arterial extracorporeal membrane oxygenation; VIS, vasoactive-inotropic score.

**Table 6:** Univariable and multivariable Cox regression analysis for survival following vaECMO removal.

	Univariable HR (95% CI)	Univariable *p*-Value	Multivariable HR (95% CI)	Multivariable *p*-Value
**Age, years**	1.045 (1.006–1.086)	**0.025**	1.048 (1.006–1.091)	**0.024**
**SAPS II at admission**	1.001 (0.969–1.034)	0.955		
**VIS at time of echocardiography**	1.032 (0.994–1.071)	0.102		
**Inotropic agents at time of echocardiography**	1.963 (0.873–4.415)	0.105		
**VIS at time of vaECMO removal**	1.029 (1.003–1.055)	**0.027**	1.030 (1.004–1.056)	**0.025**
**Inotropic agents at time of vaECMO removal**	0.503 (0.199–1.270)	0.148		
**Dobutamine dose at the time of vaECMO removal**	1.232 (1.036–1.466)	**0.029**		

Significant *p*-values are highlighted in bold. CI, confidence interval; HR, hazard ratio; SAPS, Simplified Acute Physiology Score, vaECMO, veno-arterial extracorporeal membrane oxygenation; VIS, vasoactive-inotropic score.

## Data Availability

All data included in this study are available from the corresponding author on reasonable request and in accordance with German privacy regulations.

## Abbreviations

CI	confidence interval
EF	ejection fraction
GLS	global longitudinal strain
HR	hazard ratio
ICU	intensive care unit
LV	left ventricle/left ventricular
OR	odds ratio
RV	right ventricle/right ventricular
SAPS	Simplified Acute Physiology Score
vaECMO	veno-arterial extracorporeal membrane oxygenation
VIS	vasoactive-inotropic score
